# Radiographic Evaluation of First Tarsometatarsal Joint Arthrodesis for Hallux Valgus Deformity: Does the Fusion of the First to the Second Metatarsal Base Reduce the Radiological Recurrence Rate?

**DOI:** 10.1177/19386400231164209

**Published:** 2023-05-06

**Authors:** Christian B. Scheele, Christian Kinast, Florian Lenze, Julia Wimmer, Simone Beischl, Norbert Harrasser

**Affiliations:** Department of Orthopedics and Sports Orthopedics, Technical University Munich, Klinikum rechts der Isar, Munich, Germany; Department of Orthopedics and Sports Orthopedics, Technical University Munich, Klinikum rechts der Isar, Munich, Germany; Department of Orthopedics and Sports Orthopedics, Technical University Munich, Klinikum rechts der Isar, Munich, Germany; Department of Orthopedics and Sports Orthopedics, Technical University Munich, Klinikum rechts der Isar, Munich, Germany; Department of Orthopedics and Sports Orthopedics, Technical University Munich, Klinikum rechts der Isar, Munich, Germany; Department of Orthopedics and Sports Orthopedics, Technical University Munich, Klinikum rechts der Isar, Munich, Germany

**Keywords:** hallux valgus, lapidus arthrodesis, TMT-I arthrodesis, screw positioning, intermetatarsal fusion

## Abstract

**Background:**

Modified Lapidus arthrodesis (MLA) is a well-established treatment option for symptomatic hallux valgus deformity (HVD). However, recurrence of the deformity remains a concern. The goal of this study was to evaluate the effect of an additional intermetatarsal fusion on the radiographic recurrence rate after first tarsometatarsal (TMT-I) arthrodesis.

**Methods:**

This is a retrospective evaluation of 56 feet that underwent TMT-I arthrodesis for moderate to severe HVD. Twenty-three feet received an isolated arthrodesis of the TMT-I joint (TMT-I), whereas 33 feet received an additional fusion between the base of the first and the second metatarsal bone (TMT-I/II). Various radiological parameters were determined preoperatively, 6 weeks and at a mean of 2 years postoperatively.

**Results:**

The intermetatarsal angle (IMA) and the hallux valgus angle (HVA) were significantly lowered at both follow-up evaluations in both groups. In the TMT-I/II group, the initial reduction of HVA was significantly higher (29.3° vs 21.1°). This difference disappeared by the second follow-up, leaving no significant differences between both techniques at final follow-up. Radiological recurrence rates of HVD were comparable in both groups.

**Conclusions:**

Isolated TMT-I arthrodesis provides reliable radiological results in the correction of HVD. Whether additional fusion of the first and second metatarsal base should be routinely performed remains unclear.

**Levels of Evidence:**

Level 3

“The MLA has become a well-established treatment modality for moderate to severe HVD leading to a patient satisfaction between 74% and 96%.”

## Introduction

With a prevalence of 23% to 38% hallux valgus deformity (HVD) represents the most common surgically corrected deformity of the foot.^1-4^ Relapse of the deformity is one of the most common problems after HVD correction with rates reported up to 25%.^5,6^ The risk of recurrence depends on several factors, among which the choice of surgical technique with its inherent capacity for reduction plays an important role. In this context, fusion of the first tarsometatarsal (TMT-I) joint, also known as modified Lapidus arthrodesis (MLA), is a well-established treatment modality for symptomatic HVD with low recurrence rates and good clinical outcomes.^7-9^ While Lapidus performed the arthrodesis of the first TMT-I joint without rigid internal fixation, the MLA is nowadays fixed with screws and/or plates. Even with a fused TMT-I, recurrence of metatarsus primus varus and/or HVD is still possible. Therefore, reports seeking additional intermetatarsal or intercuneiform stabilization have gained increasing attention in recent years.^10-14^ To improve stability and to reduce recurrence of intermetatarsal instability, an intermetatarsal screw from the base of the first metatarsal to the base of the second metatarsal or the middle/lateral cuneiform has been suggested by various authors.^11,15-19^ However, radiological studies concerning the outcome of intermetatarsal fusion by interposition of autogenous bone and an additional screw from the base of the first to the base of the second metatarsal are still rare and to the best of our knowledge are based only on case series.^15,20^

Therefore, the goal of this retrospective study was to compare various radiographic parameters after MLA with and without additional fusion of the first and second metatarsal base. We hypothesized that additional fixation of the first and second ray might lower the overall recurrence rates of HVD.

## Methods

The study design received approval by the Institutional Review Board and the local Ethics Committee. We retrospectively enrolled 56 patients who had painful moderate-to-severe HVD and were treated with the MLA in 2017 at 2 orthopaedic institutions, both performing more than 400 foot and ankle procedures per year (Table 1). The indication for surgical treatment was based on both clinical and radiological examination. Concerning the latter, an intermetatarsal angle (IMA) of 16° to 20° and/or a hallux-valgus-angle (HVA) of 20° to 40° were considered a moderate and an IMA > 20° and/or a HVA > 40° were considered a severe HVD.^4,21^ Recurrence of deformity was defined as IMA > 10° and HVD > 20° after final follow-up. All patients suffered from pain in the affected area, and no surgery was performed for aesthetic reasons only. Exclusion criteria were previous operations for HVD, additional correction of hindfoot pathologies during the hallux valgus surgery or lack of preoperative radiographs under full weight-bearing in 2 planes. Patients were matched to 2 groups depending on the chosen procedure: isolated fusion of the TMT-I joint (TMT-I) or additional fixation and fusion of the first to the second metatarsal base (TMT-I/II). The procedure and method of fixation were chosen according to surgeons preference without evaluating objective criteria such as first-to-second ray instability, the extent of HVD/IMA or bone quality.

**Table 1.** Patient Demographics and Characteristics.

	Total	TMT-I/II	TMT-I
No. of patients	56	33	23
Sex (female/male)	49/7	27/6	22/1
Side (left/right)	29/27	19/14	10/13
Age (years)	56.6 ± 11.5	57.7 ± 10.6	55.1 ± 12.9
HVD (moderate/severe)	24/32	13/20	11/12
IMA (moderate/severe)^a^	24/16	8/6	16/10

Abbreviations: IMA, intermetatarsal angle; HVD, hallux valgus deformity; TMT, tarsometatarsal.

a Sixteen out of 56 patients had IMA ≤ 15.

All surgeries were performed under general anesthesia (with additional foot block for postoperative analgesia) by 2 fellowship-trained foot and ankle surgeons.

Postoperatively, all patients were kept in a below-knee boot for 6 weeks, with partial weight-bearing for the first 3 weeks and transitioning to full weight-bearing for the subsequent 3 weeks.

The radiological assessment consisted of standard radiographs of the foot in 2 planes under full weight-bearing using a fully digital device (DRX-1 System, Carestream Health Deutschland GmbH, Germany). For the dorsoplantar radiographs, the central beam was oriented 20° obliquely anteriorly from vertical. For the mediolateral radiograph, the central beam was aligned horizontally in the middle of the foot (55 kV; 2 mAs). Examinations were performed before surgery, at 6 and 12 weeks, and at final follow-up (mean 25 months, range 18-36 months).

Analysis of standard radiographs was performed using DICOM PACS System (IDS7 PACS, Sectra AB, Linköping, Sweden). All measurements were done manually by 2 independent authors of the study on 2 separate occasions, randomizing the order of the images. Each observer made the measurements independently and was blinded to both patient identification and the other’s results.

**Figure 1.**
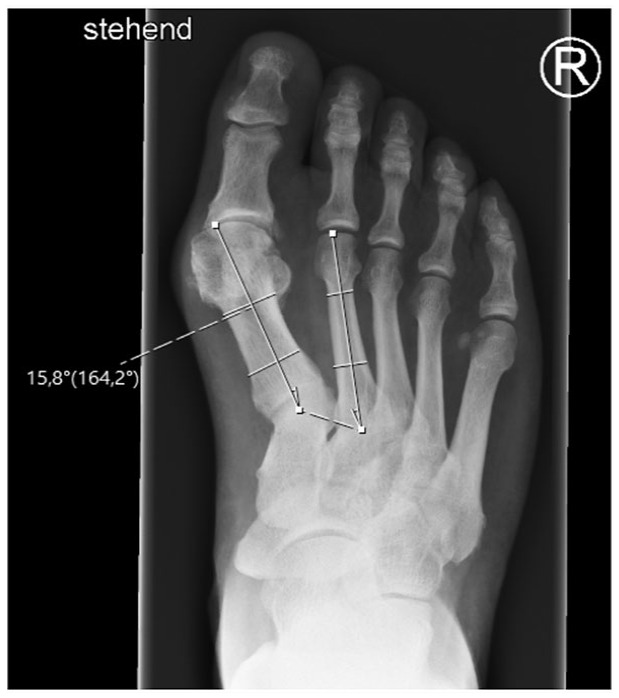
Intermetatarsal angle (IMA): Angle between the anatomical axis of the first and second metatarsal bones on a dorsoplantar radiograph in a standing position with full weight-bearing.

**Figure 2.**
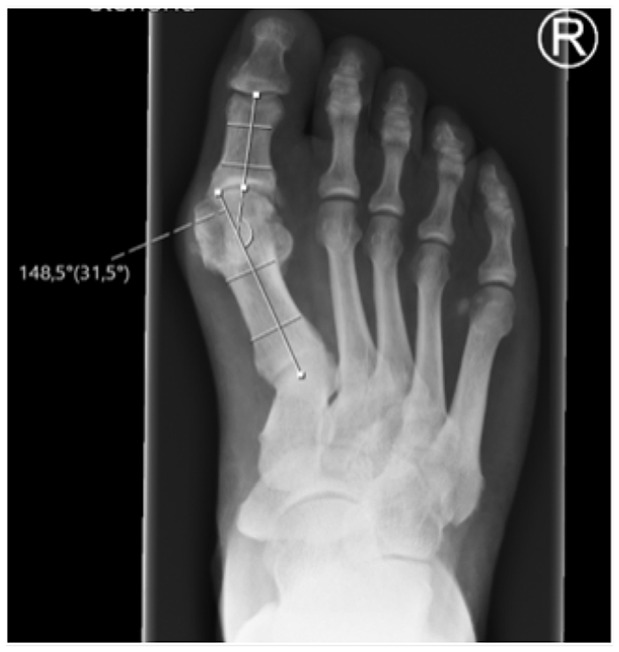
Hallux valgus angle (HVA): Angle between the anatomical axis of the first metatarsal bone and the proximal phalanx on a dorsoplantar radiograph in a standing position with full weight-bearing.

**Figure 3.**
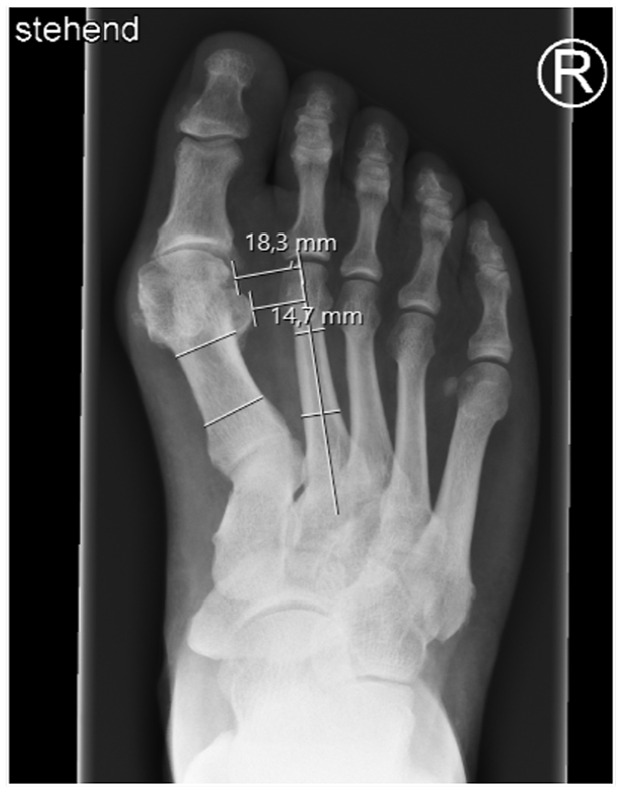
Assessment of the distance between the most lateral aspect of the head of first metatarsal and the axis of the second metatarsal (Distance MT1-MT2) and the distance of the lateral edge of the lateral sesamoid to the axis of the second metatarsal (tibial sesamoid position [TSP]) on a dorsoplantar radiograph in a standing position with full weight-bearing.

The following measurements were taken on dorsoplantar radiographs (Figure 1–3):

1. Intermetatarsal angle (IMA): Angle between the anatomical axis of the first and second metatarsal bones.
2. Hallux valgus angle (HVA): Angle between the anatomical axis of the first metatarsal bone and the proximal phalanx.
3. Distance between first and second metatarsal head (MI/II): Distance between the most lateral aspect of the head of the first metatarsal bone and the anatomical axis of the second metatarsal bone.
4. Tibial sesamoid position (TSP): Distance of the lateral edge of the lateral sesamoid bone to the anatomical axis of the second metatarsal bone.

**Figure 4.**
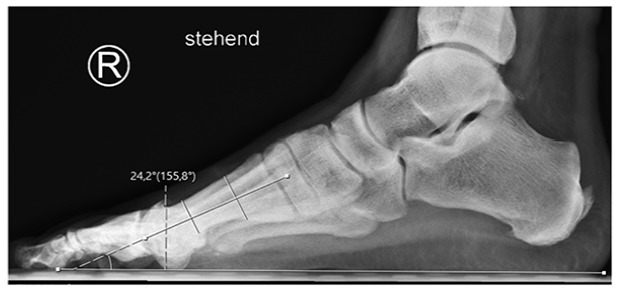
Elevation angle (ELEA) of the anatomical axis of the first metatarsal bone in relation to a line parallel to the ground assessed on a mediolateral radiograph in a standing position with full weight-bearing.

On the mediolateral radiograph, the elevation angle (ELEA) of the anatomical axis of the first metatarsal bone in relation to a line parallel to the ground was assessed (Figure 4).

### Surgical Technique

**Figure 5.**
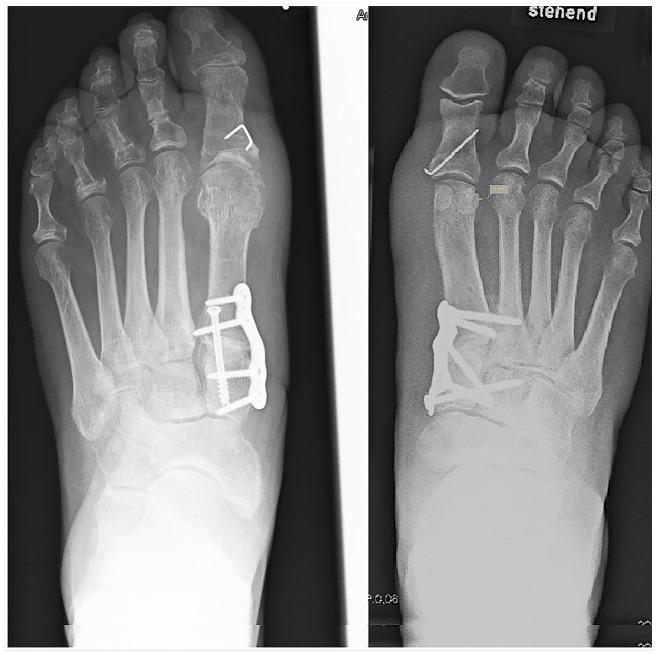
Overview of the 2 groups: Isolated fusion of the TMT-I joint (TMT-I, left) and an additional fixation and fusion of the first to the second metatarsal base (TMT-I/II, right). In this case, an additional screw in the second cuneiform was used as well.

A medial longitudinal incision from the first cuneiform to the proximal phalanx was used as the preferred approach. The first metatarsophalangeal joint was opened, the pseudoexostosis resected, and a transarticular release of the lateral joint capsule, the metatarsosesamoidal ligaments, and the adductor hallucis tendon were performed. In both methods, the TMT-I joint was prepared for fusion in a routine manner (chisel, saw), with the difference that in TMT-I/II patients, the TMT-I joint was opened with a K-wire distractor and the lateral part of the second metatarsal base was slightly decorticated with a chisel. Then autogenous cancellous bone of the previously resected pseudoexostosis was impacted between these bones. The following steps were the same for both methods. The first metatarsal was reduced to be as parallel as possible to the second metatarsal and temporarily fixed with K-wires. After fluoroscopic control, a lag screw was inserted to compress the former TMT-I joint. Depending on surgeon preference, a mediodorsal or plantar plate was used to further stabilize the TMT-I joint. In TMT-I/II patients, a screw to the second ray was passed into the base of the second metatarsal and sometimes in the second cuneiform as well (Figure 5). Finally, a medial capsuloraphy of the first metatarsophalangeal joint was carried out, and an Akin Osteotomy was added in all cases. Wound closure in a layered fashion was performed.

#### Statistics

Results are presented as absolute numbers, means, and standard deviations. Prior to analysis, Gaussian distribution was verified using Kolmogorov-Smirnoff testing. Differences between the means and all other parameters in both groups were determined using analysis of variance (ANOVA). A Bonferroni correction was used as a post-hoc test (*P* < .05: significant). The gender distribution and severity of HVD was analyzed using the chi-square test. The interobserver reliability was determined using Pearson correlation analysis, the intraobserver reliability was assessed with ICC (Intraclass Correlation Coefficient). Interobserver and intraobserver reliability was classified as minimal (correlation coefficient (CC) ≤ 0.25), low (0.26 < CC < 0.5), moderate (0.5 ≤ CC < 0.7), high (0.7 ≤ CC < 0.9), and excellent (CC ≥ 0.9).^22^ All analyses were performed with GraphPad Prism 5 (GraphPad Software, Inc., La Jolla, USA).

## Results

Both groups were comparable with respect to age, sex and, except for MI/II, severity of deformity (Table 1).

All TMT-I and TMT-I/II procedures had fused radiographically at final follow-up, complete hardware removal was observed in 4 patients. Recurrence of deformity was observed in 5 patients in the TMT-I/II group and 4 in the TMT-I group. Compared with the preoperative situation, both HVA and IMA were P=0,090 reduced in both groups and at both follow-up examinations (Table 2 to 4). At first follow-up, the HVA was significantly lower in the TMT-I/II group than in the TMT-I group (12.0° vs 20.0°). However, as the HVA increased significantly between the 2 follow-up examinations in the TMT-I/II group (12.0° vs 15.5°), there was no significant difference in HVA between the 2 groups at final follow-up (15.5° vs 18.0°). In contrast, the correction of the IMA was maintained in both groups.

**Table 2.** Perioperative Changes in TMT-I/II Group.

Parameter	Pre-OP	First follow-up	*P* value (pre-OP vs first FU)	Second follow-up	*P* value (pre-OP vs second FU)	*P* value (first vs second FU)
IMA	18.2 ± 3.6	10.1 ± 3.5	*P* <0.001	11.1 ± 3.0	*P* <0.001	*P* =0.722
HVA	41.3 ± 7.3	12.0 ± 9.5	*P* <0.001	15.5 ± 9.4	*P* <0.001	*P* =0.090
MI/II	25.9 ± 3.1	14.8 ± 2.8	*P* <0.001	16.8 ± 2.9	*P* <0.001	*P* <0.001
TSP	13.9 ± 1.9	11.1 ± 2.1	*P* <0.001	10.7 ± 2.5	*P* <0.001	*P* =1.000
ELEA	16.0 ± 4.8	19.0 ± 4.6	*P* =0.009	19.6 ± 4.0	*P* =0.003	*P* =1.000

Abbreviations: IMA, intermetatarsal angle; HVA, hallux valgus angle; MI/II, Distance between first and second metatarsal head; TSP, tibial sesamoid position; ELEA, elevation angle.

**Table 3.** Perioperative Changes in TMT-I Group.

Parameter	Pre-OP	First follow-up	*P* value (pre-OP vs first FU)	Second follow-up	*P* value (pre-OP vs second FU)	*P*-value (first vs second FU)
IMA	17.1 ± 5.1	10.5 ± 4.2	*P* =0.005	9.7 ± 3.6	*P* =0.002	*P* =1.000
HVA	41.1 ± 9.0	20.0 ± 11.8	*P* <0.001	18.0 ± 12.8	*P* <0.001	*P* =1.000
MI/II	22.6 ± 4.9	15.7 ± 3.4	*P* <0.001	15.7 ± 3.9	*P* =0.003	*P* =0.579
TSP	13.0 ± 2.1	12.2 ± 1.6	*P* =0.961	11.6 ± 2.2	*P* =0.570	*P* =1.000
ELEA	16.2 ± 3.0	16.7 ± 4.0	*P* =0.292	16.9 ± 5.4	*P* =1.000	*P* =1.000

Abbreviations: IMA, intermetatarsal angle; HVA, hallux valgus angle; MI/II, Distance between first and secondmetatarsal head; TSP, tibial sesamoid position; ELEA, elevation angle.

**Table 4.** Comparison Between Both Groups at First and Second Follow-Up Examination.

First follow-up	TMT-I/II		TMT-I		*P*-value	
	First FU	Second FU	First FU	Second FU	First FU	Second FU
IMA	10.1 ± 3.5	11.1 ± 3.0	10.5 ± 4.2	9.7 ± 3.6	*P* =0.690	*P* =0.207
HVA	12.0 ± 9.5	15.5 ± 9.4	20.0 ± 11.8	18.0 ± 12.8	*P* =0.009	*P* =0.493
MI/MII	14.8 ± 2.8	16.8 ± 2.9	15.7 ± 3.4	15.7 ± 3.9	*P* =0.325	*P* =0.318
TSP	11.1 ± 2.1	10.7 ± 2.5	12.2 ± 1.6	11.6 ± 2.2	*P* =0.041	*P* =0.271
ELEA	19.0 ± 4.6	19.6 ± 4.0	16.7 ± 4.0	16.6 ± 5.4	*P* =0.061	*P* =0.073

Abbreviations: IMA, intermetatarsal angle; HVA, hallux valgus angle; MI/II, Distance between first and second metatarsal head; TSP, tibial sesamoid position; ELEA, elevation angle.

The mean distance between the first and second metatarsal (MI/II) after surgery was significantly lowered in both groups at both follow-up examinations; significant differences between the groups were not detectable (Table 2 to 4). In contrast to the TMT-I group, the distance increased significantly between both follow-ups in the TMT-I/II group (14.8 vs 16.8).

The elevation angle (ELEA) was significantly increased from 16.0° to 19.0° at first follow-up in the TMT-I/II group. This alteration was maintained at the second follow-up. No significant changes were detectable in the TMT-I group throughout the observation period (Table 2 to 4).

The reliability testing (Table 5) for all parameters revealed high to excellent reproducibility of the measurements.

**Table 5.** Intra-/Interobserver Reliability Testing (Pearson Correlation Coefficient).

Parameters	Intraobserver reliability	Interobserver reliability
IMA	0.89	0.85
HVA	0.91	0.87
MT1-MT2	0.87	0.85
TSP (SB-MT2)	0.88	0.83
Elev. angle (°)	0.79	0.89

Abbreviations: HVA, hallux valgus angle; IMA, intermetatarsal angle; TSP, tibial sesamoid position.

## Discussion

In this study, the radiological results of 2 different fixation methods of the MLA (with and without additional fusion of the first to second metatarsal base) were evaluated after a 2-year follow-up period. Despite satisfactory correction rates and no significant revalgization angles of the hallux in both groups, no relevant effect on the recurrence rate of HVD or metatarsus primus varus was found for an additional fusion of the metatarsal bases 1 and 2.

The MLA has become a well-established treatment modality for moderate to severe HVD leading to a patient satisfaction between 74% and 96%.^7-9,23^ Various techniques for screw placement have been described, with a TMT-I joint crossing compression screw in conjunction with a plate usually being the preferred method.^20,24-26^ Despite adequate fusion of the TMT-I joint, HVD recurrence after MLA is described in 6% to 16% of cases at long-term follow-up.^27^ In this context, recurrence of an adducted first metatarsal might play a crucial role. Here, relevant intercuneiform or intermetatarsal instability has been pointed out as the major etiologic factor causing metatarsal adduction and consequently relapse of HVD.^11^ To prevent this secondary loss of correction, some surgeons perform an additional fixation between the first and second ray to achieve further stability.^10,11,13,28^ Several techniques have been described in this regard: Screw placement between first and second cuneiform, a “Lisfranc screw” from the first cuneiform to the second metatarsal base or between the base of the first and second metatarsal bones (Table 6).^10,13,29^

**Table 6.** Published Studies With Additional Screw Fixation of MLA to Intermediate (C2)/Lateral (C3) Cuneiforms or Base of Second Metatarsal (M2); M1: First Metatarsal.

Author	Design	Number of patients (feet)	Screw orientation	Follow-up (Min, Max)	Results	Complications
Bednarz and Manoli^17^	Retrospective case series	26 (31)	C1 → M1 and M1 → M2	46 months (24, 66)	19 no pain, 21 happy with foot shape HVA: 27° → 17°, IMA: 18° → 8°, elevation angle: 21° → 23°	5× recurrence HV, 2× metatarsalgia, 1× DVT, 1× hardware failure
Coetzee and Wickum^15^	Prospective case series	91 (105)	C1 → M1 and M1 → M2	3.7 Jahre (1.5, 6.2)	AOFAS Score: 52 → 81 85.5% satisfied with surgery HVA: 37.1° → 16.0°, IMA: 18° → 7.9°	7× nonunion, 5× HV recurrence, 8× hardware irritation with removal, 2× wound problems, 4× metatarsalgia
Feilmeier et al^12^	Cadaver study	12 (12)	C1 → M1 and M1 → M2/M1 → C2/C1 → C2	N/A	M1 → M2 provides more stability than others	N/A
Galli et al^13^	Cadaver study	10 (20)	2 crossing Steinmann pins (2 mm each) in TMT-I vs additional Steinmann pin M1 → C2	N/A	M1 → C2 provides more stability than TMT-I fixation only	N/A
DeVries et al^20^	Retrospective comparative analysis	143	Plate + screw (sometimes into M2 or C2/3) vs screws only (sometimes into M2 or C2/3)	Endpoint analysis	Earlier full weight-bearing with plate	nonunions: screw only (10,6%) vs plate (1,5%)
Ellington et al^16^	Retrospective case series	23	2 crossed screws TMT-I ± additional third screw M1 → M2	31.6 months (12, 60)	Differences between groups not evaluated	5× (20%) hardware irritation with removal 1× (4%) nonunion 1× (4%) recurrent HV
Habbu et al^30^	Retrospective case series	268	TMT1 ± M1 → M2(temporary)	NA	87% overall happy with outcome, differences between groups not evaluated	14× (3.8%) nonunion 4× (1.6%) hallux varus 7× (2.7%) recurrent hallux valgus
Langan et al^31^	Retrospective case series	62	Dorsomedial locking plate and screw M1 → C2 ± screw M1 → M2	9.3 months	Higher correction of IMA and HVA and less loss of correction with additional screw M1 → M2 (statistically not significant)	2× (3.2%) nonunion 4× (6.4%) recurrenct hallux valgus 3× (4.8%) hardware irritation with removal 2× (3.2%) transient neuritis
Sangeorzan and Hansen^8^	Retrospective case studies	23	2 crossed screws: C1 → M1 and M1 → C2 or M2	30 months to 6.5 years	HVA: 26° → 11° IMA: 14° → 6° 92% HVA < 15°	4× (10%) nonunion 13% revision rate

Abbreviations: HVA, hallux valgus angle; IMA, intermetatarsal angle; TSP, tibial sesamoid position.

Comparing biomechanical stability of different screw orientations in a cadaver study, *Feilmeier et al* demonstrated that the stability of the first ray after TMT-I arthrodesis can be consistently improved by an additional screw between the first and second metatarsal.^12^ Intermediate gain of stability is possible if the screw is placed from the first metatarsal into the second cuneiform (confirmed by the study of *Galli et al*^13^), and no relevant stability is obtained if the screw is placed from the first to the second cuneiform. However, these biomechanical findings seem reasonable, but so far clinical studies are rare. *Sangeorzan et al* and *Coetzee et al* were one of the first to use an intermetatarsal screw to enhance the rigidity of the Lapidus arthrodesis and to treat intercuneiform instability.^8,32^ *Fleming et al*^11^ reported intercuneiform instability in more than 70% of their Lapidus procedures treated with intermetatarsal screw fixation. These authors found few complications with their concept of targeting instability between the first 2 rays. Nevertheless, it seems worth mentioning that the placement of an intermetatarsal screw is not without risk, as the deep peroneal nerve and the dorsalis pedis artery are located close to the TMT-I.^33,34^ *So et al*^35^ identified that the intermetatarsal screw traversed the neurovascular bundle in 1 out of 10 specimens. In the remaining 9 specimens, the neurovascular bundle was located 7.1 ± 3.3 mm dorsal to the screw. Although this complication appears to be of little clinical relevance, the risk of injury to the neurovascular bundle should be considered in clinical decision-making.

In our study, we tried to substantiate the concept of intermetatarsal fusion in conjunction with the MLA by including a control group without additional fixation of the first to the second metatarsal (TMT-I group). Radiological success of the surgical procedure was demonstrated using established radiological indicators for both groups: IMA and HVA were reduced at final follow-up. These findings are in accordance with previous studies evaluating the success of the modified Lapidus procedure.^7,8,16,36,37^ Interestingly, our results indicate that the additional intermetatarsal screw fixation with fusion of the first and second metatarsal base results in a higher initial reduction of HVA compared to the TMT-I group (29.3° vs 21.1°). This could be at least partly explained by the compression effect of the intermetatarsal screw alone because all patients in both groups received an additional Akin osteotomy and a medial capsuloraphy of the first metatarsophalangeal joint. However, due to a higher loss of correction in the TMT-I/II group in terms of the distance between the first and second metatarsal (MTI/II) and HVA, this difference was not maintained until the second follow-up. Hence, no significant differences between the 2 surgical techniques were detectable at the end. Therefore, these radiological results would not confirm any benefit of an additional intermetatarsal fusion. In this context, loss of correction after MLA has been described by several authors, and risk factors have also been identified.^5,6^ Even though some authors found constant long-lasting reduction rates of HVD after intermetatarsal fusion, our findings did not support this benefit.^15^

To evaluate stability in the sagittal plane, the ELEA of the first metatarsal was measured. We found no significant changes of the ELEA from the first to second follow-up in both groups, except for a minimal increase in the TMT-I/II group after the operation. This indicates a slight plantarization of the first metatarsal in this group. However, it can be stated that both methods in this study provided stable fixation of the most distal aspect of the medial longitudinal arch, independent of intermetatarsal fusion.

The main limitations of our study include the retrospective design and small number of patients. However, most relevant studies with similar questions do have comparable patient numbers (Table 5); nevertheless, studies with higher numbers are needed to confirm our findings. Another weakness of our investigation is the lack of clinical data regarding the outcome of our patients. Even though it is generally accepted that the reduction of HVD correlates with clinical outcome in most cases, we did not collect clinical outcome data in our cohort. Nevertheless, this has to do with form and function. If correction of HVD is incomplete, limitations in hallux function are likely to persist. Another drawback of our study is the mean follow-up of 2 years, which allows only medium-term conclusions. However, previous studies have demonstrated that the greatest loss of correction occurs within the first year and remains rather constant thereafter.^27,38^

In conclusion, this study does not support the theory that an additional screw and concomitant fusion between the first and second metatarsal base can generally reduce the loss of correction or improve midterm radiographic success after MLA. In the clinical setting, some authors take an individual decision whether to place the additional intermetatarsal screw or not after an intraoperative clinical assessment of hypermobility between the MT-I and MT-II. Nevertheless, the study cannot provide the statement that an additional screw is entirely unnecessary, as recurrence of metatarsus primus varus or HVD can still occur. Further high-quality studies are needed to provide more information on this topic.

## References

[1] Arbab D Schneider L-M Schunur C Bouillon B Eysel P Konig DP. [Treatment of hallux valgus: current diagnostic testing and surgical treatment performed by German foot and ankle surgeons]. Z Orthop Unfall. 2018;156(2):193-199. 29126340 10.1055/s-0043-120352

[bibr2-19386400231164209] Nix S Smith M Vicenzino B. Prevalence of hallux valgus in the general population: a systematic review and meta-analysis. J Foot Ankle Res. 2010;3:21. 20868524 10.1186/1757-1146-3-21 PMC2955707

[bibr3-19386400231164209] Waizy H Panahi B Dohle J Stukenurg-Colsman C. The current S2e guideline for hallux valgus: evidence-based guideline development using meta-analysis. Z Orthop Unfall. 2019;157(1):75-82. 29969809 10.1055/a-0623-2966

[4] Waizy H. Hallux Valgus. DGOOC DGFOUOC. 2014. https://register.awmf.org/assets/guidelines/033-018l_S2e_Hallux_Valgus_2014-04_abgelaufen_02.pdf

[5] Raikin SM Miller AG Daniel J. Recurrence of hallux valgus: a review. Foot Ankle Clin. 2014;19(2):259-274. 24878414 10.1016/j.fcl.2014.02.008

[6] Park CH Lee WC. Recurrence of hallux valgus can be predicted from immediate postoperative non-weight-bearing radiographs. J Bone Joint Surg Am. 2017;99(14):1190-1197. 28719558 10.2106/JBJS.16.00980

[7] McInnes BD Bouche RT. Critical evaluation of the modified Lapidus procedure. J Foot Ankle Surg. 2001;40(2):71-90. 11324674 10.1016/s1067-2516(01)80048-x

[bibr8-19386400231164209] Sangeorzan BJ Hansen ST Jr. Modified Lapidus procedure for hallux valgus. Foot Ankle. 1989;9(6):262-266. 2744666 10.1177/107110078900900602

[9] Thompson IM Bohay DR Anderson JG. Fusion rate of first tarsometatarsal arthrodesis in the modified Lapidus procedure and flatfoot reconstruction. Foot Ankle Int. 2005;26(9):698-703. 16174499 10.1177/107110070502600906

[10] Plaass C Claaßen L Ettinger S Daniilidis K Stukenborg-Colsman C. [Lapidus arthrodesis]. Orthopade. 2017;46(5):424-433. 28361194 10.1007/s00132-017-3411-9

[bibr11-19386400231164209] Fleming JJ Kwaadu KY Brinkley JC Ozuzu Y. Intraoperative evaluation of medial intercuneiform instability after Lapidus arthrodesis: intercuneiform hook test. J Foot Ankle Surg. 2015;54(3):464-472. 25681280 10.1053/j.jfas.2014.12.019

[bibr12-19386400231164209] Feilmeier M Dayton P Kauwe M Cifaldi A. Comparison of transverse and coronal plane stability at the first tarsal-metatarsal joint with multiple screw orientations. Foot Ankle Spec. 2017;10(2):104-108. 27595852 10.1177/1938640016666920

[13] Galli MM McAlister JE Berlet GC Hyer CF. Enhanced Lapidus arthrodesis: crossed screw technique with middle cuneiform fixation further reduces sagittal mobility. J Foot Ankle Surg. 2015;54(3):437-440. 25456344 10.1053/j.jfas.2014.10.008

[14] Prissel MA Hyer CF Grambart ST Bussewitz BW , et al. A multicenter, retrospective study of early weightbearing for modified Lapidus arthrodesis. J Foot Ankle Surg. 2016;55(2):226-229. 26763868 10.1053/j.jfas.2015.09.003

[15] Coetzee JC Wickum D. The Lapidus procedure: a prospective cohort outcome study. Foot Ankle Int. 2004;25(8):526-531. 15363372 10.1177/107110070402500803

[bibr16-19386400231164209] Ellington JK Myerson MS Coetzee JC Stone RM. The use of the Lapidus procedure for recurrent hallux valgus. Foot Ankle Int. 2011;32(7):674-680. 21972761 10.3113/FAI.2011.0674

[bibr17-19386400231164209] Bednarz PA Manoli A 2nd . Modified lapidus procedure for the treatment of hypermobile hallux valgus. Foot Ankle Int. 2000;21(10):816-821. 11128011 10.1177/107110070002101004

[bibr18-19386400231164209] Willegger M Holinka J Ristl R Wanivenhaus AH Windhager R Schuh R. Correction power and complications of first tarsometatarsal joint arthrodesis for hallux valgus deformity. Int Orthop. 2015;39(3):467-476. 25431215 10.1007/s00264-014-2601-x

[19] Hamilton GA Mullins S Schuberth JM Rush SM Ford L. Revision Lapidus arthrodesis: rate of union in 17 cases. J Foot Ankle Surg. 2007;46(6):447-450. 17980841 10.1053/j.jfas.2007.08.005

[20] DeVries JG Granata JD Hyer CF. Fixation of first tarsometatarsal arthrodesis: a retrospective comparative cohort of two techniques. Foot Ankle Int. 2011;32(2):158-162. 21288415 10.3113/FAI.2011.0158

[21] Coughlin MJ Carlson RE. Treatment of hallux valgus with an increased distal metatarsal articular angle: evaluation of double and triple first ray osteotomies. Foot Ankle Int. 1999;20(12):762-770. 10609703 10.1177/107110079902001202

[22] Munro BH. Statistical Methods for Health Care Research. 5th ed. Philadelphia, PA: Lippincott Williams & Wilkins. pp. 248-249. 2005.

[23] Schmid T Krause F. The modified Lapidus fusion. Foot Ankle Clin. 2014;19(2):223-233. 24878411 10.1016/j.fcl.2014.02.005

[24] Ben-Ad R. Fixation updates for hallux valgus correction. Clin Podiatr Med Surg. 2014;31(2):265-279. 24685192 10.1016/j.cpm.2013.12.008

[bibr25-19386400231164209] Klos K Gueorguiev B Mückleyet T , et al. Stability of medial locking plate and compression screw versus two crossed screws for lapidus arthrodesis. Foot Ankle Int. 2010;31(2):158-163. 20132754 10.3113/FAI.2010.0158

[26] Gruber F Sinkov VS Bae S-Y Parks BG Schon LC. Crossed 1screws versus dorsomedial locking plate with compression screw for first metatarsocuneiform arthrodesis: a cadaver study. Foot Ankle Int. 2008;29(9):927-930. 18778673 10.3113/FAI.2008.0927

[27] Faber FW van Kampen PM Bloembergen MW. Long-term results of the Hohmann and Lapidus procedure for the correction of hallux valgus: a prospective, randomised trial with eight- to 11-year follow-up involving 101 feet. Bone Joint J. 2013;95-(9):1222-1226. 23997136 10.1302/0301-620X.95B9.31560

[28] Roling BA Christensen JC Johnson CH. Biomechanics of the first ray. Part IV: the effect of selected medial column arthrodeses: a three-dimensional kinematic analysis in a cadaver model. J Foot Ankle Surg. 2002;41(5):278-285. 12400710 10.1016/s1067-2516(02)80045-x

[29] Ray RG Ching RP Christensen JC Hansen ST Jr. Biomechanical analysis of the first metatarsocuneiform arthrodesis. J Foot Ankle Surg. 1998;37(5):376-385. 9798168 10.1016/s1067-2516(98)80045-8

[30] Habbu R Holthusen SM Anderson JG Bohay DR. Operative correction of arch collapse with forefoot deformity: a retrospective analysis of outcomes. Foot Ankle Int. 2011;32(8):764-773. 22049862 10.3113/FAI.2011.0764

[31] Langan TM Greschner JM Brandão RA Goss DA Jr Smith CN Hyer CF. Maintenance of correction of the modified Lapidus procedure with a first metatarsal to intermediate cuneiform cross-screw technique. Foot Ankle Int. 2020;41(4):428-436. 31878798 10.1177/1071100719895268

[32] Coetzee JC Resig SG Kuskowski M Saleh KJ. The Lapidus procedure as salvage after failed surgical treatment of hallux valgus: a prospective cohort study. J Bone Joint Surg Am. 2003;85(1):60-65. 12533573 10.2106/00004623-200301000-00010

[33] Lawrence SJ Botte MJ. The deep peroneal nerve in the foot and ankle: an anatomic study. Foot Ankle Int. 1995;16(11):724-728. 8589813 10.1177/107110079501601110

[34] Kim JW Choi Y-J Lee H-J Yi K-H Kim H-J Hu K-S. Anatomic study of the dorsalis pedis artery, first metatarsal artery, and second metatarsal bone for mandibular reconstruction. J Oral Maxillofac Surg. 2015;73(8):1627-1636. 25930957 10.1016/j.joms.2015.02.007

[35] So E Van Dyke B McGann MR Brandao R Larson D Hyer CF . Structures at risk from an intermetatarsal screw for lapidus bunionectomy: a cadaveric study. J Foot Ankle Surg. 2019;58(1):62-65. 30448378 10.1053/j.jfas.2018.08.010

[36] Orthner EHSG . [Lapidusfusion with an unidirectional plate with locking screws and immediate full weight bearing: a prospective study]. Fuß & Sprunggelenk. 2009;7(3):178-185.

[37] Olms K Braemer A Randt T Radigk S Schulz AP. Die Lapidusarthrodese zur Korrektur des Hallux valgus. Fuß Und Sprunggelenk. 2009;7:164-172.

[38] Haas Z Hamilton G Sundstrom D Ford L. Maintenance of correction of first metatarsal closing base wedge osteotomies versus modified Lapidus arthrodesis for moderate to severe hallux valgus deformity. J Foot Ankle Surg. 2007;46(5):358-365. 17761320 10.1053/j.jfas.2007.05.008

